# First Epidemiological Survey on the Prevalence and Subtypes Distribution of the Enteric Parasite *Blastocystis* sp. in Vietnam

**DOI:** 10.3390/microorganisms11030731

**Published:** 2023-03-12

**Authors:** Linh Do Ngoc Nguyen, Nausicaa Gantois, Trung Thanh Hoang, Bong Thi Do, Jeremy Desramaut, Doaa Naguib, Tuan Ngoc Tran, Anh Duc Truong, Gaël Even, Gabriela Certad, Magali Chabé, Eric Viscogliosi

**Affiliations:** 1 Family Hospital, 73 Nguyen Huu Tho Street, Da Nang 550000, Vietnam; 2 Univ. Lille, CNRS, Inserm, CHU Lille, Institut Pasteur de Lille, U1019–UMR 9017–CIIL–Centre d’Infection et d’Immunité de Lille, F-59000 Lille, France; 3 Department of Hygiene and Zoonoses, Faculty of Veterinary Medicine, Mansoura University, Mansoura 35516, Egypt; 4 GD Biotech-Gènes Diffusion, F-59000 Lille, France; 5 PEGASE-Biosciences (Plateforme d’Expertises Génomiques Appliquées aux Sciences Expérimentales), Institut Pasteur de Lille, F-59000 Lille, France; 6 Délégation à la Recherche Clinique et à l’Innovation, Groupement des Hôpitaux de l’Institut Catholique de Lille, F-59000 Lille, France

**Keywords:** *Blastocystis* sp., intestinal protozoa, Southeast Asia, Vietnam, molecular epidemiology, transmission, zoonosis

## Abstract

Although *Blastocystis* sp. is the most common enteric protozoan in human stools worldwide, various geographical areas remain to be investigated regarding the frequency and circulation of this parasite. Such is the case of some developing countries in Southeast Asia that exhibit a higher risk for parasitic infections due to unsanitary conditions. While several epidemiological surveys have been conducted, for instance, in Thailand, little or no data are available from neighboring countries, such as Vietnam. Therefore, in order to determine the prevalence and subtype (ST) distribution of *Blastocystis* sp. and to clarify the transmission of the parasite, the first molecular epidemiological survey ever conducted in this country was performed. For this purpose, a total of 310 stool specimens were collected from patients enrolled at the Family Hospital of Da Nang and then tested for the presence of *Blastocystis* sp. by real-time Polymerase Chain Reaction (qPCR), followed by subtyping of the isolates. The overall prevalence of the parasite reached 34.5% in this Vietnamese cohort. No significant association was found between parasite infection and gender, age, symptomatic status, contact with animals or source of drinking water. Out of the 107 positive patients, nearly half presented mixed infections. Therefore, some of the corresponding samples were reanalyzed by end-point PCR, followed by PCR products cloning and sequencing. Of the 88 total subtyped isolates, ST3 was predominant, followed by ST10, ST14, ST7, ST1, ST4, ST6 and ST8. Our study was, thus, the first to report ST8, ST10 and ST14 in the Southeast Asian population. The predominance of ST3 within this Vietnamese cohort, coupled with its low intra-ST genetic variability, reflected a large inter-human transmission, while ST1 transmission was suggested to be not only anthroponotic, but also likely correlated to animal or environmental sources. Strikingly, isolates considered of animal origin (ST6-ST8, ST10 and ST14) accounted for more than 50% of the subtyped isolates. These findings improved our knowledge of the epidemiology and circulation of *Blastocystis* sp. in Southeast Asia, and in particular, in Vietnam, and highlighted both a major burden of the parasite in this country and a high risk of zoonotic transmission, mainly from poultry and livestock.

## 1. Introduction

*Blastocystis* sp. is currently the most common intestinal protozoan identified in the human population worldwide [1,2,3,4] since its prevalence has often been reported to exceed 50% in developing countries from diverse geographical areas and, in particular, in the African continent [5,6,7]. This enteric microorganism has also been frequently detected in the gastrointestinal tract of a wide range of animals, including numerous groups of mammals, birds, fish, reptiles and insects [8,9,10,11]. Like other intestinal protozoa, the main mode of transmission of *Blastocystis* sp. is the indirect fecal–oral route through the consumption of water and eventually food contaminated by *resistant cystic forms* of the parasite excreted by various hosts [2,12,13,14]. Transmission can also occur through direct contact, either with infected humans or animals, due to the zoonotic potential of this microorganism [15]. Based on its observed frequency, an estimated 1 billion people could be infected with *Blastocystis* sp. across the world, with a large majority of them asymptomatic carriers [16]. In this respect, the presence of this parasite is generally associated with a healthy gut microbiota, according to repeated metagenomics surveys conducted in colonized humans [17,18,19,20]. However, under particular circumstances, a proportion of individuals vulnerable to this parasitic infection and its pathogenicity presents gastrointestinal symptoms, mainly diarrhea and abdominal pain, and even skin disorders, such as urticaria [12,21,22]. Moreover, several findings suggest that colorectal cancer may be related with elevated risk of *Blastocystis* sp. infection [23]. Recent in vitro studies, coupled with genomic data, resulted in the identification of different virulence factors involved in the pathophysiology of this parasite, which could be associated with the genetic diversity of *Blastocystis* sp. isolates [24,25].

It has been evidenced that the genus *Blastocystis* presents a wide genetic diversity, based on the comparison of the *small subunit ribosomal RNA* (*SSU rRNA*) gene sequences. Accordingly, 38 genetic variants, called subtypes (STs), that may correspond to species have been proposed at this time (ST1 to ST38) [26,27]. Among these STs, 34 of them have been validated, while the remaining 4 STs (ST18-ST20 and ST22) could represent chimaeras arising during Polymerase Chain Reaction (PCR) amplification [28]. Among all these STs, only 14 (ST1-ST10, ST12, ST14, ST16 and ST23) have been found in the human population, with extremely variable frequencies [7,29,30,31,32,33]. Indeed, by globally analyzing the epidemiological data available in numerous countries, ST1 to ST4 represent more than 90% of the subtyped human isolates [29]. Accordingly, and even if these four STs can be found in animal groups, ST1 to ST4 are, thus, essentially transmitted by the inter-human route. Conversely, other STs found less frequently or rarely in humans are considered to be of animal origin because of their predominance in particular groups of mammals or birds. For example, ST5 mainly occurs in pigs, ST6 and ST7 in poultry and ST10 and ST14 in livestock [8,9,10]. The presence of these STs in the human population can, therefore, be explained by zoonotic transmission, as evidenced in recent studies through the identification of isolates exhibiting the same *SSU rRNA* gene sequence in both individuals and in-contact animals [15,34,35].

Because of the potential impact of *Blastocystis* sp. on public health and the growing use of PCR-based approaches for its detection, a noticeable increasing interest for this enteric protozoan has been recorded over the last decade with the completion of a very large number of epidemiological studies worldwide. However, some geographical areas remain under-investigated, like that of Southeast Asia. Indeed, while numerous surveys have been performed in Thailand, and to a lesser extent in Malaysia, Indonesia and the Philippines, as recently reviewed [1,2], little or no epidemiological data are available for other southeast Asian countries, such as Vietnam. However, parts of this lower-middle-income country with a population of nearly 100 million people are at high risk of parasitic infections, mainly due to a lack of water sanitation. To date, only two studies concerning the frequency of intestinal parasites in Vietnamese individuals are found in the literature [36,37]. Nevertheless, both studies, conducted outside Vietnam, involved two cohorts of Vietnamese immigrants arriving in Taiwan. The prevalence of *Blastocystis* sp. observed in these two surveys was similar and around 20%. However, the real prevalence was probably underestimated, considering that the parasite was only identified by direct-light microscopy observation of fecal smears, a low-sensitivity diagnostic method for the detection of *Blastocystis* sp. compared to PCR assays [38,39].

Therefore, the first molecular epidemiological survey ever performed in Vietnam was conducted on a cohort of more than 300 patients followed at the Family Hospital of Da Nang. The aim of the present study was, thus, to determine the prevalence and ST distribution of *Blastocystis* sp. isolates identified in this Vietnamese cohort by real-time PCR (qPCR) and sequencing, then to identify potential modes of transmission of the parasite within this population according to subtyping data.

## 2. Materials and Methods

### 2.1. Ethics Approval and Questionnaire

The present study was approved by the Research Ethics Committee of Family Hospital of Da Nang, Vietnam (reference number 201120/QD-FAMILY; Date of approval: 20 November 2020). This survey was conducted in accordance with the Code of Ethics of the World Medical Association (Declaration of Helsinki III) and with the International Ethical Guidelines for Biological Research Involving Human Subjects. The research objectives of the study were clearly explained to all the participants prior to enrollment, and written consent forms were obtained from each adult or from the parents of minors. Each research participant was provided with a carefully designed questionnaire to collect epidemiological information, including sex, age, place of residence, contact with animals, source of drinking water (well, tap or mineral water) and presence of gastrointestinal symptoms (diarrhea, abdominal pain, vomiting, abdominal bloating and constipation). If at least one of the 5 selected digestive disorders listed above was reported, the individual was considered symptomatic. The data obtained for each subject were fully anonymized through the encryption of the identity of individuals.

### 2.2. Sampling Site and Collection of Samples

**Figure 1:**
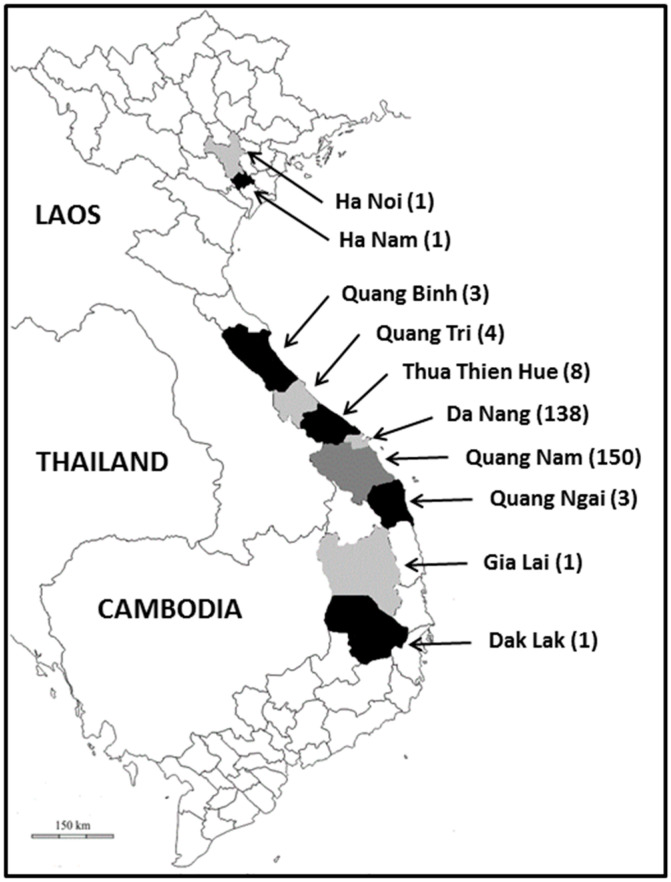
Map of Vietnam showing the place of residence of the subjects enrolled in this survey. The number of participants living in each province or municipality is indicated between brackets.

Briefly, Vietnam, Southeast Asia, is a country divided into 58 provinces and 5 municipalities corresponding to the 5 most populated cities of the country. Da Nang (geographical coordinates: latitude 16°3′16″ N, longitude 108°12′8″ E), which is located in the Central Coast region of southern Vietnam, is the fourth largest city of the country, with an estimated population of around 1 million people (Figure 1). Da Nang borders the South China Sea and is located about 600 km from Hanoi, the capital, and Ho Chi Minh City. It is also one of the most important port cities in Vietnam. All the samples were collected between December 2020 and October 2021 in the Family Hospital located in the North of the Da Nang agglomeration.

A total of 310 patients, followed for different pathologies or routine examinations and presenting or not digestive disorders, were enrolled. These patients were originally from 10 provinces or municipalities in Vietnam, but the large majority of them lived in the Da Nang municipality (138/310, 44.5%) and the neighboring province of Quang Nam (150/310, 48.4%) (Figure 1).

Stool samples (1 sample per patient) were collected during routine clinical procedures. For each subject, approximately 2 g of fresh stool was added to 2 mL of 2.5% potassium dichromate (*w*/*v* in water) (Sigma Life Sciences, Saint Louis, MO, USA) in a sterile Falcon tube, then mixed vigorously for homogenization before storage at 4 °C. The samples were subsequently transferred to the Pasteur Institute of Lille (France) for further processing.

### 2.3. DNA Extraction, PCR Assays and Molecular Subtyping/Genotyping of Blastocystis sp. Isolates

Upon arrival in France, the fecal specimens were washed 3 times with distilled water by centrifugation at 3000 g for 10 min to remove any trace of potassium dichromate. The last supernatant was discarded, and the resulting ultimate pellet was diluted in 500 µL of sterile water. The total genomic DNA was extracted from the diluted pellet using the NucleoSpin 96 Soil kit or NucleoSpin Soil, Mini kit for DNA from Soil (Macherey-Nagel GmbH & Co KG, Düren, Germany), following the recommended procedures of the manufacturer. DNA was eluted in 100 μL of elution buffer provided in the kits and stored at −20 °C. The *Blastocystis*-specific primers BL18SPPF1 (5′-AGTAGTCATACGCTCGTCTCAAA-3′) and BL18SR2PP (5′-TCTTCGTTACCCGTTACTGC-3′) were used to amplify the *SSU rRNA* gene by qPCR from 2 μL of extracted DNA, as previously described [39]. The corresponding amplified gene domain of about 300 bp has been shown to contain sufficient sequence information for accurate subtyping of *Blastocystis* sp. isolates. PCR screening was conducted in duplicate for each sample, and positive (*Blastocystis* sp. ST8 DNA obtained from axenic culture) and negative (reagent-grade water) controls were included in the assays. The STs of *Blastocystis* sp.-positive specimens were identified by sequence analysis of the purified qPCR products (Genoscreen, Lille, France; SANGER technology platform, 3730XL DNA Analyzer). For a significant proportion of samples, a double trace was observed during the analysis of the sequence chromatogram, suggesting mixed infection by at least two different *Blastocystis* sp. STs. To provide insight into the distribution of STs in mixed infections, some specimens were selected based on the qPCR amplification intensity for reanalysis by end-point PCR, using the same primer pair as for qPCR, as previously detailed [40]. The PCR products obtained and visualized on agarose gel were purified using the NucleoSpin Gel and PCR Clean-up kit (Macherey-Nagel GmbH & Co KG), cloned in the T-vector TA pCR–TOPO 2.1 of the TOPO TA cloning kit (Invitrogen, Carlsbad, CA, USA) following the manufacturer’s protocol, then amplified in One Shot TOP10 Chemically Competent *Escherichia coli* (Invitrogen). Minipreparations of plasmids were performed using the NucleoSpin Plasmid kit (Macherey-Nagel GmbH & Co KG). For each specimen, five positive clones exhibiting an insert of the expected size were randomly selected and sequenced on both strands. The obtained sequences in this study have been deposited in GenBank under the accession numbers OP716077-OP716164. For subtyping of the isolates, the obtained SSU rRNA sequences were compared with all homologous sequences available for the known *Blastocystis* sp. STs at the National Centre for Biotechnology Information (NCBI), using the nucleotide Basic Local Alignment Search Tool (BLAST) program. The sequences of *Blastocystis* sp. isolates from the same ST were subsequently aligned against each other using the BioEdit v7.0.1 package (Date of release 10 June 2019; http://www.mbio.ncsu.edu/BioEdit/bioedit.html, accessed on 1 February 2022), with the aim to assess the intra-ST diversity and identify the so-called genotypes, as previously described [6,7,31].

### 2.4. Statistical Analysis

Fisher’s exact test was used to study the potential relationship between the various category variables. *Blastocystis* sp. colonization and STs were chosen as the main outcomes, and the multilevel logistic mixed regression models were created to generate odds ratios (ORs) and the 95% confidence interval (CI). The *p*-value of less than 0.05 was set as the limit for significance. All analyses were conducted using the package statistics and odds ratio 2.0.1 from the R statistical computing program (Version 4.1.1 Date of release 8 October 2021; R Development Core Team, http://www.R—project.org, accessed on 1 May 2022).

## 3. Results and Discussion

To our knowledge, this study is the first investigation of the molecular epidemiology of *Blastocystis* sp. ever conducted in the Vietnamese population. In this context, the stool samples of a cohort of 310 patients enrolled at the Family Hospital in Da Nang and aged 7 months to 89 years (mean age of 34 ± 12 years) were screened for the presence of this parasite by qPCR. Of the 310 specimens tested, 80 were positive, for an overall prevalence of 34.5% (Table 1).

This frequency was, thus, approximately doubled compared to that previously reported in Vietnamese immigrants in Taiwan using non-molecular methods [36,37]. On the other hand, and considering only investigations performed in southeast Asia using molecular detection assays, the prevalence in Vietnam was close to the average frequency reported from neighboring countries, such as Thailand (22.3%), Indonesia (31.8%) or Malaysia (24.7%), but significantly lower than those observed in the Philippines (49.1%) and Cambodia (55.2%), even if this frequency was based on a single epidemiological study for the latter country [1,2]. Moreover, in some of these countries, for which a relatively large number of surveys have been conducted, such as Thailand, the Philippines and Malaysia, the prevalence observed varies greatly depending on the geographical area or the cohort considered, ranging from 5.2 to 40.6%, 15.3 to 82.9% and 9.2 to 40.3%, respectively [1,23]. All these data clearly highlight the active circulation of *Blastocystis* sp. in Vietnam and more globally in the Southeast Asian population.

Statistical tests were performed, focusing on the cohort of patients from the Da Nang municipality (138 individuals) and Quang Nam province (150 subjects), which together accounted for around 93% (288/310) of the participants included in this study. Considering these 2 most frequent places of residence, no significant association between parasite infection and geographical area was found (35.5% of positive individuals in Da Nang versus 34.7% in Quang Nam; Fisher exact test, *p* = 0.902). Within this cohort of 288 subjects, neither gender (Fisher exact test, *p* = 0.137), age (Fisher exact test, *p* = 0.486) and contact with animals (Fisher exact test, *p* = 0.242) nor consumption of well water (Fisher exact test, *p* = 0.372) were significantly associated with the infection. The prevalence of *Blastocystis* sp. was also not significantly higher in patients considered symptomatic (15.8%) versus asymptomatic subjects (11.2%) (Fisher exact test, *p* = 0.273). Additionally, none of the digestive disorders considered in this survey was significantly more frequent in symptomatic patients, such as the case of diarrhea (Fisher exact test, *p* = 0.204), abdominal pain (Fisher exact test, *p* = 0.328), constipation (Fisher exact test, *p* = 0.389) or vomiting (Fisher exact test, *p* = 0.126). None of the subjects indicated suffering from abdominal bloating in the present study.

Direct sequencing of the 107 purified qPCR products from positive samples resulted in the identification of 55 single infections with a broad range of STs (Table 1). ST3 was the major reported ST (n = 23, 41.9%), followed by ST10 (n = 8, 14.6%), ST7 (n = 7, 12.7%), ST1 and ST14 (n = 6 each, 10.9%), ST4 and ST6 (n = 2 each, 3.6%) and ST8 (n = 1, 1.8%) (Table 1). The remaining positive samples (52/107, 48.6%) corresponded to mixed infections, according to the analysis of sequence chromatograms. Such a high rate of mixed infections in the Vietnamese cohort likely reflected numerous sources of *Blastocystis* sp. infection, whether human, animal or environmental. To clarify this issue, 13 samples selected from the 52 corresponding to mixed infections were re-analyzed by end-point PCR, before the cloning of PCR products and sequencing of corresponding clones. Six mixed infections by two STs (two samples with ST10/ST14 and one sample with ST1/ST7, ST3/ST10, ST3/ST14 or ST7/ST14) and seven mixed infections by three STs (five samples with ST3/ST10/ST14 and one sample with either ST3/ST7/ST14 or ST3/ST7/ST10) were recorded. Not surprisingly, these mixed infections corresponded to different mixtures of the four STs (ST3, ST7, ST10 and ST14) occurring with the highest frequencies in our cohort. With the addition of these new data, a total of 88 subtyped isolates were considered (Table 2).

Among them, ST3 remained largely predominant, representing 36.4% (n = 32) of the isolates, followed by ST10 (n = 17), ST14 (n = 16), ST7 (n = 11), ST1 (n = 7), ST4 and ST6 (n = 2 each) and ST8 (n = 1) (Table 2). Based on these epidemiological data, no significant difference in the ST distribution was revealed according to age, sex of the participants and contact with animals. Interestingly, this ST distribution varied widely between the Da Nang municipality and Quang Nam province. Indeed, a higher prevalence of ST1 infection was reported in Da Nang (*p* = 0.055), while some other STs were overrepresented in Quang Nam province, including ST7 (*p* = 0.320), ST10 (*p* = 0.056) and ST14 (*p* = 0.060), even if these associations were not significant. By grouping the bovine ST10 and ST14 isolates, persons living in the Quang Nam area had a 3.4 higher risk of infection by these isolates, compared to persons from Da Nang (OR: 3.400, CI: 1.302–10.583, *p* = 0.019). This difference in ST distribution could be explained by the rurality of the province of Quang Nam in comparison to the more urbanized Da Nang area and the increased presence in the vicinity of dwellings of poultry and livestock, which are known to be carriers of ST7 and ST10/ST14 [8,9,10], respectively, and thus potential reservoirs of parasite transmission to inhabitants. Moreover, individuals who drank well water (OR: 10.875, CI: 1.770–68.146, *p* = 0.008) or presented constipation (OR: 11.375, CI: 1.311–81.393, *p* = 0.016) were significantly predictive of the risk of ST1 infection.

By comparing this global ST distribution in Vietnam with those documented in other Southeast Asian countries [1,2], ST3 was also predominant in the Philippines, Malaysia and Cambodia, as described in a majority of countries worldwide, while in contrast, ST1 was most common in Thailand and Indonesia. Strikingly, the distribution of STs in Vietnam presented a major particularity, with a large proportion of isolates considered as zoonotic. Indeed, the avian ST6 and ST7, the ST8 known to colonize primates as other hosts, and the bovine-adapted ST10 and ST14 [2,8,9,10,35] accounted for more than 50% of the subtyped isolates, highlighting a wide zoonotic transmission of *Blastocystis* sp. in this Vietnamese cohort. Furthermore, while ST6, ST7 or both STs had already been recognized in communities from Thailand, the Philippines, Indonesia and Malaysia, the present study was the first to identify ST8, ST10 and ST14 in the Southeast Asian population investigated so far [1,2].

**Figure 2:**
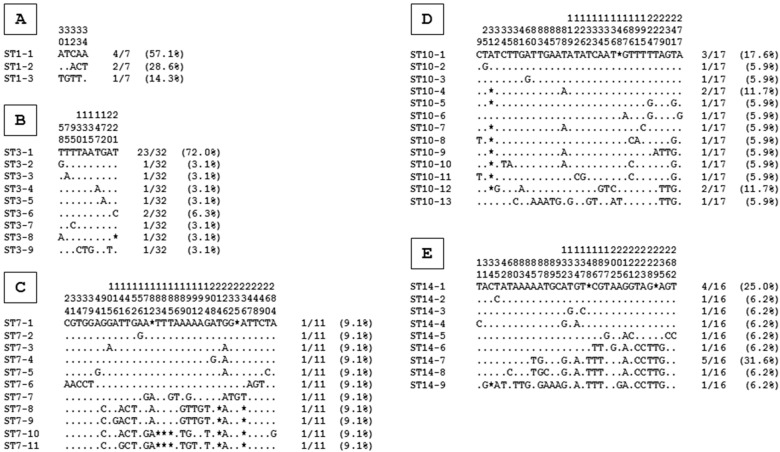
Alignment of partial *SSU rRNA* gene sequences from *Blastocystis* sp. ST1 (**A**), ST3 (**B**), ST7 (**C**), ST10 (**D**) and ST14 (**E**) isolates. Only the variable positions are shown in these alignments. The positions of variable positions with respect to the reference sequences (genotypes ST1-1, ST3-1, ST7-1, ST10-1 and ST14-1) are indicated above the alignment (vertical numbering). Nucleotides identical to those of the reference sequences are represented by dashes, and gaps are represented by asterisks. All the genotypes identified for each ST are indicated on the left of the alignment, whereas the total number and percentage of isolates identified in our study for each genotype are reported on the right.

To expand our knowledge of the circulation of *Blastocystis* sp. in Vietnam, sequences of isolates belonging to the main STs were aligned with each other to evaluate intra-ST diversity and identify so-called genotypes (Figure 2).

Briefly, the analysis of the variable positions within the alignments of each ST resulted in the identification of 3 genotypes of ST1, for a total of 7 isolates and, thus, an average ratio of 2.3 isolates per genotype. The average ratios observed for the other STs were 3.6 for ST3 (32 isolates for 9 genotypes), 1 for ST7 (11 isolates for 11 genotypes), 1.3 for ST10 (17 isolates for 13 genotypes) and 1.8 for ST14 (16 isolates for 9 genotypes). Since this ratio is inversely proportional to intra-ST variability, ST3 exhibited the lowest intra-ST diversity, followed by ST1, ST14, ST10 and ST7. Using the same approach, the lowest sequence variability among ST3 isolates compared to ST1 was also described in surveys conducted in Guinea [7], Senegal [6] and Lebanon [41]. The low number of ST3 genotypes could be undoubtedly correlated to active human-to-human transmission within the Vietnamese population since this ST is not frequently reported in domestic or farm animals. Moreover, the circulation of the predominant ST3-1 genotype (72% of the ST3 isolates) in both the Da Nang and Quang Nam areas reinforced this statement. ST1 transmission could also be in large part anthroponotic regarding the low number of ST1 genotypes, even if this ST was identified in different animal groups and as the most widespread ST from various water sources in Asiatic countries [2]. Consequently, a wide transmission of certain ST1 genotypes to the population through the consumption of water contaminated by feces from various hosts could not be excluded. Interestingly, the impressive number of genotypes identified for ST7 was inconsistent with large-scale transmission from limited sources of contamination. Although the majority of participants colonized with ST7 did not report contact with animals, they were likely individually infected after contact with bird droppings in water or food, as, for instance, in vegetable gardens or markets. A similar hypothesis could be put forward to explain the huge number of bovine ST10 and ST14 genotypes found in the Vietnamese population. The large amount of free-range and farmed animals in rural Vietnam, coupled with poor water sanitation in some areas, could actively contribute to the spreading of *Blastocystis* sp. zoonotic STs. In this respect, ST10 was, for instance, identified in various water sources in a neighboring country, Malaysia [41].

Moving forward, a multi-center epidemiological study has to be performed in southern Vietnam, with the aim of confirming the active circulation of *Blastocystis* sp. in this country and its transmission patterns suggested from data collected in the Da Nang area. Additionally, and for a better understanding of parasite transmission dynamics, it remains crucial to explore *Blastocystis* sp. under the One Health perspective by screening human, animal and environmental samples collected within the same restricted Vietnamese geographical area, as it was recently conducted in a rural community in northern Thailand [33].

## Figures and Tables

**Table 1:** Prevalence and ST distribution of *Blastocystis* sp. in the 10 Vietnamese provinces and municipalities screened in the present study.

				*Blastocystis* sp. STs								
Provinces/ Municipalities	Samples (n)	Positive Samples (n)	Prevalence (%)	ST1	ST3	ST4	ST6	ST7	ST8	ST10	ST14	MI ^a^
Da Nang	138	49	35.5	5	14	0	1	2	1	1	1	24
Quang Nam	150	52	34.7	1	9	2	1	5	0	6	5	23
Thua Thien Hué	8	2	25.0	0	0	0	0	0	0	0	0	2
Quang Tri	4	0	0	-	-	-	-	-	-	-	-	-
Quang Ngai	3	1	33.3	0	0	0	0	0	0	0	0	1
Quang Binh	3	2	66.7	0	0	0	0	0	0	1	0	1
Dak Lak	1	0	0	-	-	-	-	-	-	-	-	-
Gia Lai	1	1	100	0	0	0	0	0	0	0	0	1
Ha Nam	1	0	0	-	-	-	-	-	-	-	-	-
Ha Noi	1	0	0	-	-	-	-	-	-	-	-	-
**Total**	**310**	**107**	**34.5**	**6**	**23**	**2**	**2**	**7**	**1**	**8**	**6**	**52**

^a^ MI, Mixed infections.

**Table 2:** Distribution of *Blastocystis* sp. STs in the Vietnamese cohort after considering subtyped isolates present in mixed infections.

	*Blastocystis* sp. STs								
Provinces/ Municipalities	ST1	ST3	ST4	ST6	ST7	ST8	ST10	ST14	MI ^a^
Da Nang	6	18	0	1	3	1	4	4	19
Quang Nam	1	14	2	1	7	0	12	11	16
Thua Thien Hué	0	0	0	0	0	0	0	0	2
Quang Tri	-	-	-	-	-	-	-	-	-
Quang Ngai	0	0	0	0	1	0	0	1	0
Quang Binh	0	0	0	0	0	0	1	0	1
Dak Lak	-	-	-	-	-	-	-	-	-
Gia Lai	0	0	0	0	0	0	0	0	1
Ha Nam	-	-	-	-	-	-	-	-	-
Ha Noi	-	-	-	-	-	-	-	-	-
**Total**	**7**	**32**	**2**	**2**	**11**	**1**	**17**	**16**	**39**

^a^ MI, Mixed infections.

## Data Availability

Not applicable.
